# Comparison of Functional Recovery and In-Hospital Outcomes Between Thrombolysed and Non-thrombolysed Patients With Acute Ischemic Stroke: A Retrospective Comparative Cohort Study

**DOI:** 10.7759/cureus.111966

**Published:** 2026-07-02

**Authors:** Muhammad Aftab Ahmad, Nizar Haddad, Muhammad Uzair Israr, Azmeer Aamir, Anum Karamat Baig, Iman Ikram, Ashir Saleem

**Affiliations:** 1 Internal Medicine, King Edward Medical University, Lahore, PAK; 2 Internal Medicine, Basildon University Hospital, Basildon, GBR; 3 Psychiatry, Sheikh Zayed Hospital, Rahim Yar Khan, PAK; 4 Internal Medicine, Mercy Teaching Hospital, Peshawar, PAK; 5 Internal Medicine, Jinnah Hospital, Lahore, PAK; 6 Internal Medicine, Shalamar Hospital, Lahore, PAK

**Keywords:** acute ischemic stroke, intracranial hemorrhage, modified rankin scale, national institutes of health stroke scale, thrombolysed

## Abstract

Background

Acute ischemic stroke is a major cause of mortality and long-term disability worldwide, contributing substantially to healthcare costs and socioeconomic burden, particularly in low- and middle-income countries. Timely restoration of cerebral perfusion remains the cornerstone of acute stroke management and is associated with improved neurological recovery and functional outcomes.

Objective

This study’s objective is to compare functional recovery, in-hospital mortality, complications, and duration of hospitalization between thrombolysed and non-thrombolysed patients with acute ischemic stroke.

Methods

This retrospective comparative cohort study was conducted at Shalamar Hospital, Lahore, Pakistan, from December 2022 to December 2025 and included 157 patients diagnosed with acute ischemic stroke. All eligible patients meeting the inclusion criteria during the study period were included through non-probability consecutive sampling. Patients were categorized into thrombolysed (n = 62) and non-thrombolysed (n = 95) groups. Baseline stroke severity was assessed using the National Institutes of Health Stroke Scale (NIHSS), and functional outcomes were evaluated with the Modified Rankin Scale (mRS). The primary outcome was functional status at discharge.

Results

Thrombolysed patients had higher baseline NIHSS scores (13.2 ± 4.1 vs. 11.5 ± 3.8, p = 0.011). Despite greater initial severity, a higher proportion achieved favorable functional outcomes (mRS 0-2) compared to non-thrombolysed patients (37 (59.7%) vs. 38 (40.0%) (p = 0.014)). In-hospital mortality was lower in the thrombolysed group (six (9.7%) vs. 18 (18.9%) (p = 0.048)). Symptomatic intracranial hemorrhage occurred more frequently among thrombolysed patients (five (8.1%) vs. two (2.1%) (p = 0.041)). Mean hospital stay was shorter in the thrombolysed group (6.8 ± 2.9 days vs. 8.4 ± 3.7 days, p = 0.003).

Conclusion

Intravenous thrombolysis was associated with improved functional recovery, lower in-hospital mortality, and shorter hospitalization compared with conservative management. Although thrombolysis was associated with a higher frequency of symptomatic intracranial hemorrhage, the overall findings suggest a favorable benefit-risk profile in appropriately selected patients.

## Introduction

Acute ischemic stroke (AIS) remains one of the leading causes of mortality and long-term disability worldwide, with a substantial socioeconomic burden on healthcare systems, particularly in low- and middle-income countries [1]. It accounts for approximately 85% of all stroke cases and is defined by a sudden interruption of cerebral blood flow due to arterial occlusion, resulting in cerebral ischemia and neuronal infarction [2]. Despite advances in preventive strategies, the incidence of ischemic stroke continues to rise, particularly among the ageing population, where risk factors, such as hypertension, diabetes mellitus, dyslipidemia, atrial fibrillation, and sedentary lifestyles, are increasingly prevalent [3].

Cerebral ischemia initiates a cascade of biochemical events, including excitotoxicity, oxidative stress, mitochondrial dysfunction, and inflammatory activation, ultimately leading to irreversible neuronal injury. Surrounding the infarct core is the ischemic penumbra, a region of hypoperfused but potentially salvageable tissue [4]. The primary goal of acute stroke management is the timely restoration of perfusion to this penumbral tissue to prevent irreversible damage. This concept underscores the critical importance of early intervention and provides the biological basis for thrombolytic therapy [5].

Intravenous thrombolysis using recombinant tissue plasminogen activator (rtPA), particularly alteplase, has revolutionized the management of AIS by targeting the underlying thrombotic occlusion [6]. The landmark National Institute of Neurological Disorders and Stroke (NINDS) trial demonstrated that patients treated within three hours of symptom onset had significantly improved functional outcomes at three months compared to placebo [7]. Subsequent trials, including ECASS III and IST-3, extended the therapeutic window up to 4.5 hours in selected patients and supported its use in older populations. However, strict eligibility criteria are essential to minimize treatment-related risks [8]. The most serious complication is symptomatic intracranial hemorrhage, necessitating careful patient selection and rapid clinical and radiological evaluation to balance the benefits of reperfusion against the risk of hemorrhagic transformation [9].

In routine clinical practice, a considerable proportion of patients with AIS do not receive thrombolytic therapy [10]. Common reasons include delayed hospital presentation beyond the therapeutic window, unclear time of symptom onset, contraindications such as recent surgery or active bleeding, uncontrolled hypertension, and limited access to specialized stroke care [11]. Additionally, pre-hospital delays, lack of public awareness, transportation barriers, and infrastructural limitations in developing healthcare systems further restrict the timely administration of thrombolysis [12]. Consequently, conservative management - including antiplatelet therapy, supportive care, and secondary prevention - remains the primary treatment modality for many patients.

Approximately 30% of patients with AIS present with mild neurological deficits (defined as National Institutes of Health Stroke Scale (NIHSS) score ≤5) in certain populations, such as in China [13], while this proportion may reach up to 50% in the United States. However, up to one-third of these patients may experience unfavorable outcomes, particularly in the presence of large vessel occlusion [14]. Notably, large vessel occlusion strokes may present with mild initial symptoms, with nearly one-third of such cases having an NIHSS score <5 at presentation [15].

Although the efficacy of intravenous thrombolysis has been established through randomized clinical trials and large international registries, real-world evidence from low- and middle-income countries remains limited. In Pakistan, delays in hospital presentation, restricted access to specialized stroke services, and variations in healthcare infrastructure may substantially influence patient selection for thrombolysis and subsequent clinical outcomes. Moreover, locally generated comparative data evaluating functional recovery, in-hospital mortality, complications, and healthcare utilization among thrombolysed and non-thrombolysed patients are scarce. Therefore, this study was undertaken to provide real-world evidence from a tertiary care center in Pakistan, which may help inform clinical practice, optimize resource allocation, and improve stroke care in similar resource-constrained settings.

Objective

This study’s objective is to compare functional recovery, in-hospital mortality, complications, and duration of hospitalization between thrombolysed and non-thrombolysed patients with AIS.

## Materials and methods

Study design and setting

This retrospective comparative cohort study was conducted at Shalamar Hospital, Lahore, Pakistan, from December 2022 to December 2025. A total of 157 patients with confirmed AIS were included.

Patients were identified through hospital electronic databases and medical record archives using diagnostic codes and neuroimaging reports. Non-probability consecutive sampling was employed, and all eligible patients admitted during the study period were included.

Based on treatment modality, patients were categorized into two groups: those who received intravenous thrombolytic therapy and those managed conservatively without thrombolysis. Patients who did not receive intravenous thrombolysis because they presented beyond the recommended therapeutic window (>4.5 hours from symptom onset) or had contraindications to thrombolytic therapy were included in the non-thrombolysed group.

Inclusion and exclusion criteria

Patients aged ≥18 years with a confirmed diagnosis of AIS on CT or MRI, complete clinical and outcome records, and admission during the study period were included irrespective of eligibility for thrombolytic therapy. Patients who presented within the therapeutic window and met institutional eligibility criteria received intravenous thrombolysis, whereas those presenting beyond the therapeutic window or with contraindications to thrombolysis were managed conservatively and included in the non-thrombolysed group.

Patients with hemorrhagic stroke on neuroimaging, transient ischemic attack (TIA), incomplete or missing medical records, or transfer to another institution before completion of management were excluded.

Data collection

A structured data collection proforma was used to retrospectively extract data from patient case files, discharge summaries, imaging reports, and electronic medical records.

Data extracted from medical records included demographic characteristics, such as age and sex, vascular risk factors, including hypertension, diabetes mellitus, dyslipidemia, atrial fibrillation, and smoking status, as well as time from symptom onset to hospital presentation. Information regarding in-hospital complications, including symptomatic intracranial hemorrhage, aspiration pneumonia, recurrent stroke, intensive care unit admission, mortality, and duration of hospitalization, was also collected and analyzed.

Collected variables included demographic characteristics (age and gender), vascular risk factors (hypertension, diabetes mellitus, dyslipidemia, atrial fibrillation, and smoking status), and time from symptom onset to hospital presentation.

Baseline stroke severity was assessed using the admission NIHSS score [9]. Functional outcomes were evaluated using the Modified Rankin Scale (mRS) at discharge [16]. A favorable outcome was defined as an mRS score 0-2, while an unfavorable outcome was defined as an mRS score 3-6 [8]. Long-term follow-up data were not consistently available and, therefore, were not included in the analysis.

Patients in the thrombolysis group received intravenous alteplase according to standard institutional stroke protocols. The non-thrombolysis group comprised patients who did not receive intravenous alteplase because they presented beyond the recommended therapeutic time window or had one or more contraindications to thrombolytic therapy according to institutional and international stroke management guidelines. Common reasons included unknown or delayed symptom onset (>4.5 hours), uncontrolled hypertension, recent major surgery or active bleeding, anticoagulant use with contraindicating coagulation parameters, extensive established infarction on neuroimaging, or other physician-determined contraindications. Patients in the non-thrombolysis group received standard medical management, including antiplatelet therapy, statins, blood pressure control, and supportive care.

The primary outcome was a favorable functional status at discharge (mRS 0-2). Secondary outcomes included in-hospital mortality, frequency of complications, and length of hospital stay.

Statistical analysis

Data were entered and analyzed using SPSS (IBM SPSS Statistics for Windows, IBM Corp., Version 26.0, Armonk, NY). Quantitative variables, including age, baseline NIHSS score, and length of hospital stay, were expressed as mean ± standard deviation. Qualitative variables, including gender, comorbidities, treatment groups, complications, and functional outcome categories, were presented as frequencies and percentages.

The independent-samples t-test was used to compare means between the two groups. The chi-square test or Fisher’s exact test was applied for comparison of categorical variables, as appropriate. A p-value of ≤0.05 was considered statistically significant.

## Results

A total of 157 patients with AIS were included, with a mean age of 63.5 ± 12.1 years. Patients in the thrombolysed group were slightly younger than those in the non-thrombolysed group (61.4 ± 11.2 vs. 64.8 ± 12.6 years). Males constituted 95 (60.5%) of the study population, with comparable distribution between groups (38 (61.3%) vs. 57 (60.0%)).

Hypertension was the most prevalent comorbidity (116 (73.9%)), followed by dyslipidemia (87 (55.4%)), diabetes mellitus (80 (51.0%)), smoking (48 (30.6%)), and atrial fibrillation (30 (19.1%)), with no significant differences between groups. Baseline stroke severity was significantly higher in the thrombolysed group, with a mean NIHSS score of 13.2 ± 4.1 compared to 11.5 ± 3.8 in the non-thrombolysed group (p = 0.011) (Table 1).

**Table 1:** Demographic and Baseline Clinical Characteristics (N = 157) Independent-samples t-test was used for continuous variables. Chi-square test was used for categorical variables. *Statistically significant at p ≤ 0.05. NIHSS: National Institutes of Health Stroke Scale [9]

Variable	Thrombolysed (n = 62)	Non-Thrombolysed (n = 95)	Total (N = 157)	Test Statistic	p-value
Age (Years), Mean ± SD	61.4 ± 11.2	64.8 ± 12.6	63.5 ± 12.1	t = -1.73	0.086
Male	38 (61.3%)	57 (60.0%)	95 (60.5%)	χ² = 0.03	0.864
Female	24 (38.7%)	38 (40.0%)	62 (39.5%)	-	-
Hypertension	44 (71.0%)	72 (75.8%)	116 (73.9%)	χ² = 0.46	0.498
Diabetes Mellitus	29 (46.8%)	51 (53.7%)	80 (51.0%)	χ² = 0.73	0.392
Dyslipidemia	33 (53.2%)	54 (56.8%)	87 (55.4%)	χ² = 0.19	0.663
Atrial Fibrillation	11 (17.7%)	19 (20.0%)	30 (19.1%)	χ² = 0.13	0.721
Smoking	18 (29.0%)	30 (31.6%)	48 (30.6%)	χ² = 0.12	0.726
Baseline NIHSS Score, Mean ± SD	13.2 ± 4.1	11.5 ± 3.8	12.2 ± 4.0	t = 2.59	0.011*

At discharge, favorable functional outcomes (mRS 0-2) were achieved in 75 (47.8%) of patients overall, with a significantly higher proportion in the thrombolysed group compared to the non-thrombolysed group (37 (59.7%) vs. 38 (40.0%) (p = 0.014)). Conversely, unfavorable outcomes (mRS 3-6) were more common in the non-thrombolysed group (57 (60.0%) vs. 25 (40.3%)).

In-hospital mortality was significantly lower in the thrombolysed group (six (9.7%) vs. 18 (18.9%) (p = 0.048)) (Table 2).

**Table 2:** Functional Outcomes at Discharge (Modified Rankin Scale) Chi-square test applied. *Statistically significant at p ≤ 0.05. mRS: Modified Rankin Scale [16]

Outcome	Thrombolysed (n = 62)	Non-Thrombolysed (n = 95)	Total (N = 157)	Test Statistic	p-value
Favorable (mRS 0-2)	37 (59.7%)	38 (40.0%)	75 (47.8%)	χ² = 6.03	0.014*
Unfavorable (mRS 3-6)	25 (40.3%)	57 (60.0%)	82 (52.2%)	-	-
In-Hospital Mortality	6 (9.7%)	18 (18.9%)	24 (15.3%)	χ² = 3.91	0.048*

Symptomatic intracranial hemorrhage occurred in seven (4.5%) of patients overall and was significantly more frequent in the thrombolysed group compared to the non-thrombolysed group (five (8.1%) vs. two (2.1%) (p = 0.041)). There were no statistically significant differences between groups in rates of aspiration pneumonia, recurrent stroke, or ICU admission.

The mean length of hospital stay was significantly shorter in the thrombolysed group (6.8 ± 2.9 days) compared to the non-thrombolysed group (8.4 ± 3.7 days, p = 0.003) (Table 3).

**Table 3:** In-Hospital Complications Chi-square test applied. Independent-samples t-test for hospital stay. *Statistically significant at p ≤ 0.05.

Complication	Thrombolysed (n = 62)	Non-Thrombolysed (n = 95)	Total (N = 157)	Test Statistic	p-value
Symptomatic Intracranial Hemorrhage	5 (8.1%)	2 (2.1%)	7 (4.5%)	Fisher’s Exact	0.041*
Aspiration Pneumonia	9 (14.5%)	19 (20.0%)	28 (17.8%)	χ² = 0.77	0.382
Recurrent Stroke	3 (4.8%)	7 (7.4%)	10 (6.4%)	Fisher’s Exact	0.506
ICU Admission	14 (22.6%)	18 (18.9%)	32 (20.4%)	χ² = 0.34	0.561
Hospital Stay (Days), Mean ± SD	6.8 ± 2.9	8.4 ± 3.7	7.8 ± 3.5	t = -3.02	0.003*

Stroke severity stratification showed that 24 (15.3%) of patients had mild stroke, 99 (63.1%) had moderate stroke, and 34 (21.7%) had severe stroke, with no significant difference between groups. Among patients with severe stroke (NIHSS >15), favorable outcomes were significantly more frequent in the thrombolysed group compared to the non-thrombolysed group (eight (47.1%) vs. three (17.6%) (p = 0.038)) (Table 4).

**Table 4:** Stroke Severity Stratification and Outcomes Chi-square test applied. *Statistically significant at p ≤ 0.05. NIHSS: National Institutes of Health Stroke Scale [9]

Variable	Thrombolysed (n = 62)	Non-Thrombolysed (n = 95)	Total (N = 157)	Test Statistic	p-value
Mild Stroke (NIHSS ≤5)	6 (9.7%)	18 (18.9%)	24 (15.3%)	χ² = 2.52	0.112
Moderate Stroke (NIHSS 6-15)	39 (62.9%)	60 (63.2%)	99 (63.1%)	χ² = 0.001	0.971
Severe Stroke (NIHSS >15)	17 (27.4%)	17 (17.9%)	34 (21.7%)	χ² = 1.96	0.162
Favorable Outcome in Severe Stroke	8 (47.1%)	3 (17.6%)	11 (32.4%)	Fisher’s Exact	0.038*

A significantly higher proportion of thrombolysed patients presented within three hours of symptom onset compared to non-thrombolysed patients (44 (71.0%) vs. 21 (22.1%) (p < 0.001)). In contrast, 58 (61.1%) of non-thrombolysed patients presented beyond 4.5 hours, rendering them ineligible for thrombolysis (p < 0.001).

The mean door-to-needle time in the thrombolysis group was 54.3 ± 12.7 minutes. Antiplatelet therapy within 24 hours was administered in 62 (100%) of thrombolysed patients and 91 (95.8%) of non-thrombolysed patients, with no significant difference between groups (p = 0.123) (Table 5).

**Table 5:** Time to Presentation and Treatment Characteristics Chi-square test applied. *Statistically significant at p ≤ 0.05.

Variable	Thrombolysed (n = 62)	Non-Thrombolysed (n = 95)	Test Statistic	p-value
Arrival within 3 Hours	44 (71.0%)	21 (22.1%)	χ² = 36.8	<0.001*
Arrival 3-4.5 Hours	18 (29.0%)	16 (16.8%)	χ² = 3.27	0.071
Arrival >4.5 Hours	0 (0%)	58 (61.1%)	Fisher’s Exact	<0.001*
Door-to-Needle Time (Minutes), Mean ± SD	54.3 ± 12.7	-	-	-
Antiplatelet Initiation Within 24 Hours	62 (100%)	91 (95.8%)	Fisher’s Exact	0.123

## Discussion

This retrospective comparative cohort study evaluated the clinical outcomes of thrombolysed and non-thrombolysed patients with AIS and demonstrated that intravenous thrombolysis was associated with significantly improved functional recovery, reduced in-hospital mortality, and shorter hospital stay. Notably, these benefits were observed despite higher baseline stroke severity in the thrombolysed group, underscoring the clinical effectiveness of timely reperfusion therapy in real-world settings.

A significant difference in baseline NIHSS scores was observed between the two groups, with higher scores in the thrombolysed cohort, indicating more severe neurological deficits at presentation [9]. Despite this, thrombolysed patients achieved a significantly higher proportion of favorable functional outcomes (59.7% vs. 40.0%) and a lower in-hospital mortality (9.7% vs. 18.9%). These findings suggest an association between thrombolysis and improved functional outcomes, even among patients presenting with relatively severe strokes. However, because of the retrospective observational design and the absence of multivariable adjustment, causality cannot be inferred. Previous studies have consistently demonstrated that early intravenous thrombolysis improves functional independence and reduces short-term mortality, particularly when administered within the recommended therapeutic window [17].

An important observation in this study was the subgroup analysis of patients with severe stroke (NIHSS >15), where thrombolysed patients exhibited significantly better outcomes compared to those managed conservatively. This finding aligns with existing literature indicating that patients with moderate to severe strokes derive substantial benefit from reperfusion therapy due to the presence of salvageable ischemic penumbra. These results challenge the clinical hesitancy often associated with administering thrombolysis in severe stroke and highlight the importance of timely decision-making [18].

As expected, symptomatic intracranial hemorrhage was more frequent in the thrombolysed group (8.1% vs. 2.1%), reflecting the known risks associated with thrombolytic therapy [19]. However, despite this increased risk, overall mortality remained lower in the thrombolysed cohort. These findings are consistent with previous evidence suggesting a favorable benefit-risk profile of thrombolysis in appropriately selected patients, although the observational nature of this study precludes definitive conclusions regarding treatment effect. The present findings reinforce the favorable efficacy-safety balance of thrombolytic therapy in routine clinical practice [20].

Another clinically relevant finding was the significantly shorter hospital stay among thrombolysed patients (6.8 ± 2.9 days vs. 8.4 ± 3.7 days). This reduction likely reflects earlier neurological recovery, improved functional status, and fewer complications. Prior studies have similarly shown that successful reperfusion is associated with faster clinical stabilization and reduced healthcare utilization [21]. In resource-limited healthcare systems, shorter hospitalization has important economic implications and may enhance healthcare capacity.

Time-to-presentation analysis further emphasized the critical role of early hospital arrival. A substantial proportion of non-thrombolysed patients presented beyond the 4.5-hour therapeutic window, rendering them ineligible for thrombolysis. This highlights persistent gaps in stroke care pathways, including delayed symptom recognition and referral. Previous literature has identified public awareness, efficient emergency medical services, and streamlined in-hospital stroke protocols as key determinants of timely thrombolysis. These findings underscore the need for targeted community education and optimization of pre-hospital and emergency response systems [22].

A major strength of this study is that it provides real-world evidence on the effectiveness of intravenous thrombolysis in a tertiary care setting in Pakistan, where published data on AIS outcomes remain limited. Unlike many previous studies conducted in highly controlled clinical trial settings, this study reflects routine clinical practice and demonstrates that thrombolysed patients achieved better functional outcomes, lower in-hospital mortality, and shorter hospital stay despite presenting with higher baseline stroke severity. These findings are consistent with landmark randomized trials and contemporary observational studies, supporting the external validity of our results while contributing important regional evidence from a low- and middle-income country. Furthermore, the inclusion of treatment timelines, stroke severity stratification using the NIHSS, functional outcome assessment with the mRS, and clinically relevant in-hospital outcomes provides a comprehensive evaluation of thrombolysis in routine clinical care. Collectively, these strengths enhance the clinical relevance of the study and provide valuable evidence to support decision-making regarding thrombolytic therapy in resource-limited healthcare settings.

Several limitations should be considered when interpreting these findings. First, the retrospective, single-center design limits the generalizability of the results and introduces the potential for information bias due to reliance on the accuracy and completeness of medical records. Second, the non-randomized design may have resulted in selection bias, as treatment allocation was based on clinical eligibility and time to presentation rather than random assignment. Furthermore, the analysis was primarily descriptive and comparative; multivariable logistic regression was not performed to adjust for potential confounding factors. Therefore, the observed differences between thrombolysed and non-thrombolysed patients should be interpreted as associations rather than independent treatment effects. Additionally, long-term functional outcomes were not available, and advanced neuroimaging parameters, such as infarct volume and collateral circulation, were not consistently documented. Prospective multicenter studies with adjustment for important confounders are needed to validate these findings.

## Conclusions

In this retrospective comparative cohort study, intravenous thrombolysis was associated with improved functional outcomes, lower in-hospital mortality, and shorter hospitalization among patients with AIS. Although thrombolysis was associated with a higher incidence of symptomatic intracranial hemorrhage, a greater proportion of thrombolysed patients achieved favorable neurological recovery compared with those managed conservatively. These findings are consistent with previous evidence supporting the effectiveness of timely reperfusion therapy in appropriately selected patients.

Given the observational nature of the study, the lack of multivariable adjustment, and the potential for residual confounding, these results should be interpreted as demonstrating an association rather than a causal treatment effect. Delayed presentation beyond the therapeutic window remained a major barrier to thrombolytic treatment, highlighting the importance of public awareness, early stroke recognition, and efficient emergency referral pathways. Further prospective multicenter studies with multivariable adjustment and long-term follow-up are warranted to validate these findings and better define the independent effect of thrombolysis on functional recovery and survival.
